# Investigation of Deformation Behavior of Additively Manufactured AISI 316L Stainless Steel with In Situ Micro-Compression Testing

**DOI:** 10.3390/ma16175980

**Published:** 2023-08-31

**Authors:** Fei Teng, Ching-Heng Shiau, Cheng Sun, Robert C. O’Brien, Michael D. McMurtrey

**Affiliations:** 1 Idaho National Laboratory, Idaho Falls, 1955 N Fremont Ave., Idaho Falls, ID 83415, USA; cheng.sun@inl.gov (C.S.); michael.mcmurtrey@inl.gov (M.D.M.); 2 Micron School of Materials Science & Engineering, Boise State University, 1910 University Drive, Boise, ID 83725, USA; 3 Center for Advanced Energy Studies, 995 University Blvd., Idaho Falls, ID 83401, USA

**Keywords:** additive manufacturing, AISI 316L stainless steel, microstructure, small-scale mechanical properties

## Abstract

Additive manufacturing techniques are being used more and more to perform the precise fabrication of engineering components with complex geometries. The heterogeneity of additively manufactured microstructures deteriorates the mechanical integrity of products. In this paper, we printed AISI 316L stainless steel using the additive manufacturing technique of laser metal deposition. Both single-phase and dual-phase substructures were formed in the grain interiors. Electron backscatter diffraction and energy-dispersive X-ray spectroscopy indicate that Si, Mo, S, Cr were enriched, while Fe was depleted along the substructure boundaries. In situ micro-compression testing was performed at room temperature along the [001] orientation. The dual-phase substructures exhibited lower yield strength and higher Young’s modulus compared with single-phase substructures. Our research provides a fundamental understanding of the relationship between the microstructure and mechanical properties of additively manufactured metallic materials. The results suggest that the uneven heat treatment in the printing process could have negative impacts on the mechanical properties due to elemental segregation.

## 1. Introduction

Additive manufacturing (AM) techniques have been applied in various fields due to the low cost and near-net-shape fabrication of complex components. A typical process of AM is to use laser to selectively melt the feedstock of powders, and the melted powders consolidate as the geometry of the components builds up layer by layer [1,2]. Several AM methods have been developed to manufacture metallic components, such as laser-beam melting (LBM), laser metal deposition (LMD) and electron-beam melting (EBM) [3]. LBM is a powder-bed-based process where metal powders are spread in thin layers on the bottom of the process chamber with a powder recoater and the laser is directed to the powder bed to melt the powders. This method has been used to fabricate austenitic stainless steels (SSs) [4,5,6,7,8,9,10,11,12,13], maraging SSs [14,15,16,17], precipitation-hardenable SSs [18,19] and martensitic cutlery SSs [20,21]. LMD feeds and melts metal powders simultaneously in the deposition head, which includes laser energy inputs and coaxial/multi-jet powder feeding nozzles. AISI 316L SS [22,23,24] and H13 SS [25] have been fabricated using this approach. Unlike LBM or EBM using laser as the heating source, EBM uses an electron beam to melt the metal powders, with a hopper feeding the powders and a rake spreading the powders [26]. EBM has been applied to fabricated tool steels and AISI 316L SS. The study of 3D-printed microstructures is of great importance to the following industrial applications, as the unique features affect the mechanical properties, manufacturing properties and surface finish, which provides more potential methods to manipulate microstructures in the right way based on the needs. Previous research shows that 316L SS made using laser powder-bed fusion consisted of a hierarchical austenitic microstructure with a fine, 300–700 nm subgrain cellular structure and a high number of dislocations. Appropriate heat treatment can reduce dislocations and increase precipitates significantly, which provides the possibility to make printed 316L SS comparable to conventional technologies [27]. Compared with conventional material extrusion, laser powder-bed fusion can also provide lower porosity and higher hardness [28]. Inconel 718 parts manufactured with the machining process of LMD are also found to be different from the forged workpiece in chip geometry and bending moment [29]. The surface finish can also be improved by optimizing the inclination angle and strategies in the process of LMD [30]. 

The additive manufacturing process determines the unique microstructure of AM products and thus the mechanical properties. Manufacturing parameters such as scanning method, laser power and energy distribution play a critical role in the development of the microstructure [31,32]. AM products typically exhibit a certain level of porosity due to the inherent pores trapped within powders [33]. A very low porosity of AISI 316L stainless steel, ~0.38%, has been reported in the literature [34]. Carlton et al. [5] studied the impact of porosity on the deformation behavior of austenitic 316L SS and pointed out that a ductile failure mode is typical of low remaining porosity (0.1%) and a brittle failure mode is usually found with high remaining porosity (2.4%). Previous studies of additively manufactured 316L SS using SLM [13,35] and laser-engineered net shape (LENS) [36] have also reported the anisotropic bulk behavior related to the printing direction. Both printing methods show that the samples tested in the direction parallel to the printing layers have higher yield strength and ultimate tensile strength than samples tested perpendicularly to the printing layers. Both homogenous single-phase (fcc-structured austenite) and dual-phase (fcc-structured austenite and bcc-structured ferrite) structures [37] were reported in additively manufactured AISI 316L SS. Yadollashi et al. [22] reported that AISI 316L SS fabricated with LMD, which is LENS, contain 9% δ-ferrite and 91% austenite, and exhibit higher yield strength and lower elongation compared with AISI 316L SS with single austenite phase fabricated with LBM [12,35]. No ferrite existing in LBM-built AISI 316L SS has been reported in previous research [12], while ferrite has been reported in previous research on LMD/LENS-built AISI 316L SS after additive manufacturing processing [22,36]. A fundamental understanding of the formation of dual-phase microstructures and corresponding mechanical responses is still unclear. 

The object of this study is to explore the impact of heterogeneous microstructures formed near the printed surface region on the corresponding mechanical stability using in situ SEM micro-pillar compression. In this paper, AISI 316L SS was fabricated with the LMD approach. The printed microstructure and microchemistry were characterized and correlated to the mechanical properties. Real-time observations of deformation failure in the [001] orientation of single-phase and dual-phase structures were performed using in situ micro-pillar compression testing with a scanning electron microscope. Our research provides new insights into the deformation behavior of AM metallic materials and into the design of AM processes for metallic materials. 

## 2. Experimental Methods

AISI 316L SS was fabricated using a laser-engineered net shape (LENS) MR-7 printer, which is based on the LMD approach, with laser energy set to 400 W, a 600 µm spot size, and a scanning speed of 12.7 mm/s in argon atmosphere without active cooling. A crosshatch scanning pattern was used, and the corresponding spacing between scanning paths was ~250 µm. Detailed information on the 316L SS powder can be found in a previous report [38]. The powder was blown with four nozzles directly into the melting pool. The specimen was built one layer at a time. To understand the effect introduced by the printing process, no extra heat treatment was performed after printing. The chemical composition of the AM316L specimen is shown in Table 1. The microstructure of the printed AISI 316L SS was characterized with scanning electron microscopy (SEM), electron backscatter diffraction (EBSD) and X-ray micro-computed tomography (micro-CT). FEI Quanta FEG 650 SEM was used to image the grain morphology and substructures. The samples were polished with 1200 grit SiC paper, 1 µm diamond suspension and 0.05 µm alumina suspension and were then electro-etched in a solution of 15.4% nanopure water + 46.1% H_3_PO_4_ and 38.5% H_2_SO_4_ at 4 V voltage at room temperature for 13 s. An FEI Quanta Helios Plasma Focused Ion Beam (PFIB) equipped with EBSD and X-ray energy-dispersive spectrum (EDS) detectors was used to map the grain orientation and chemical distribution. The samples were mechanically polished and subsequently plasma-etched. 

The specimen was removed from the print using electrical discharge machining. The coupon, approximately 3.8 × 3.8 × 24.3 mm, was characterized using X-ray computed tomography (XCT). XCT reconstructions of the specimen were acquired using a North Star Imaging Micro XCT system. The X-ray generator was a Hamamatsu 150 kV micro-focus X-ray tube (model #L12161-07) with a 7 µm nominal spot size. The detector was a Dexela 2923 NDT, a flat panel X-ray detector with 75 µm pixel pitch and an active area of 290.8 by 229.8 mm. A rotation stage between the source and detector provided precision rotation, enabling computed tomography reconstruction and showing voids larger than 10 µm.

**Figure 1:**
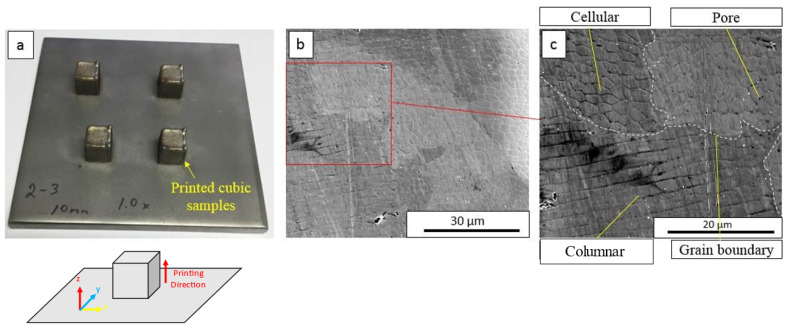
(**a**) The sketch indicates the printing orientation for the additively manufactured 316SS used in this study. (**b**) Microstructure of AM316L. (**c**) Images show grain boundary, the two existing types of substructures (cell and columnar) and pores.

In situ micro-compression tests were performed with an FEI Quanta 3D Focused Ion Beam (FIB). Micro-pillars with dimensions of 4 µm × 4 µm × 8 µm were fabricated using the same FIB. Both single-phase and dual-phase pillar grains were selected on the top surface of the printed cube shown in Figure 1. EBSD mapping was used to determine grain orientation and the corresponding phase before pillar fabrication. A flat punch tip with an area of 20 µm × 20 µm was installed on a Bruker PicoIndenter-88 platform. Compression tests were performed at room temperature along the [001] orientation for both single-phase and dual-phase structures. A constant displacement rate of 40 nm/s with total displacement of 4 µm was used during the compression tests. The microstructure of compressed pillars was characterized using a JEOL 2010 transmission electron microscope. 

## 3. Results

### 3.1. Microstructure and Microchemistry

**Figure 2:**
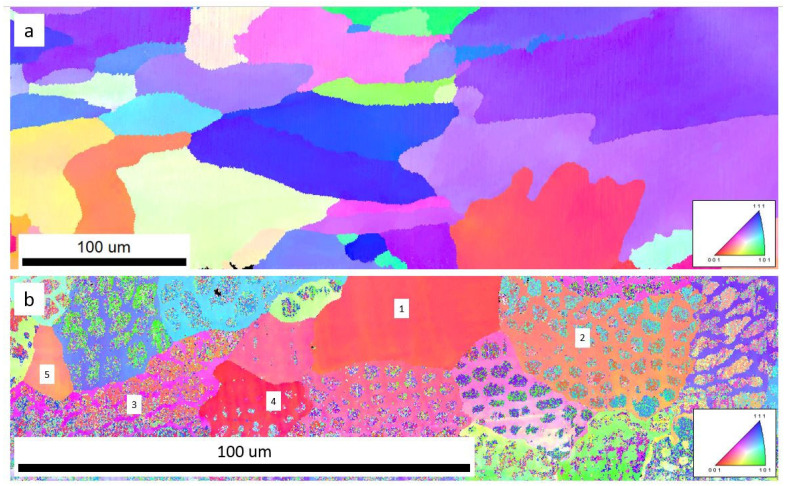
OIM of additively manufactured AISI 316L SS observed with EBSD. (**a**) Single-phase fcc-structured microstructure. The average grain size is ~100 µm. (**b**) Dual-phase microstructure with fcc-structure austenite and bcc-structured ferrite. The average grain size is ~50 µm. Grains 1 and 5 in Figure 2b are homogenous fcc-structured austenite, and grains 2, 3 and 4 are dual-phase structures.

In the as-printed AISI 316L SS, both cellular and columnar substructures in the interiors of grains were formed, as seen in the SEM micrographs in Figure 1. The average size of the cellular substructures was around 5 µm. Grain boundaries formed close to the substructure boundary (Figure 1b,c). Orientation imaging microscopy in Figure 2 shows un-uniform distribution of grain size in the microstructure. Figure 2a shows that the average grain size of the single-phase region was around 100 µm with single fcc-structured austenite phase, while in the dual-phase regions, near the edge of the specimen, as shown in Figure 2b, the average grain size was ~50 µm with both fcc-structured austenite and bcc-structured ferrite. The grains are shown to be distorted and affected by printing orientation, which makes some grains larger in a direction (EBSD mappings are attached in Supplemental Materials Figure S5). Grains 1 and 5 in Figure 2b are homogenous fcc-structured austenite, and grains 2, 3 and 4 are dual-phase structures. Pores were observed in the as-printed AISI 316L SS using SEM and X-ray micro-CT (attached in Supplemental Materials Figure S7). In Figure 1c, pores with a diameter of 1–2 µm preferentially formed along the substructure boundaries. A statistical study with ImageJ software version 1.53k shows that the average single-pore area was 0.0543 µm^2^ (average pore size was ~0.132 µm) and the porosity was 0.9585%. 

**Figure 3:**
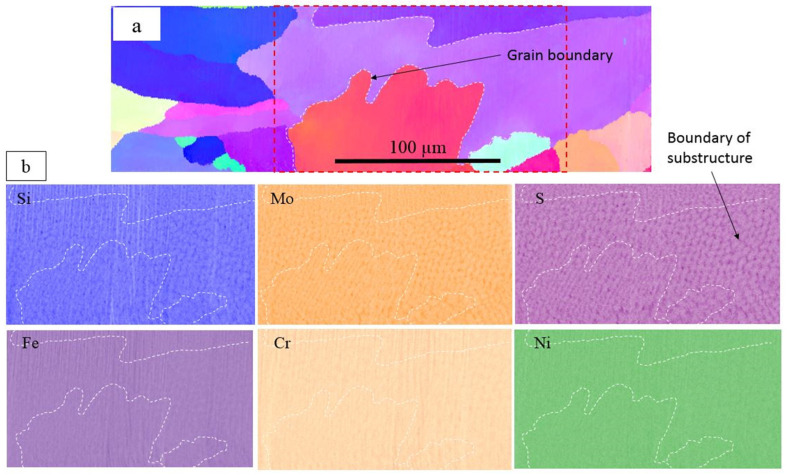
Chemical analysis of homogenous structures in as-printed AISI 316L SS. (**a**) Grain boundary and substructure boundary are labeled in EBSD mapping. (**b**) Si, Mo, S, Cr and Ni were enriched, while Fe was depleted along the substructure boundary.

**Figure 4:**
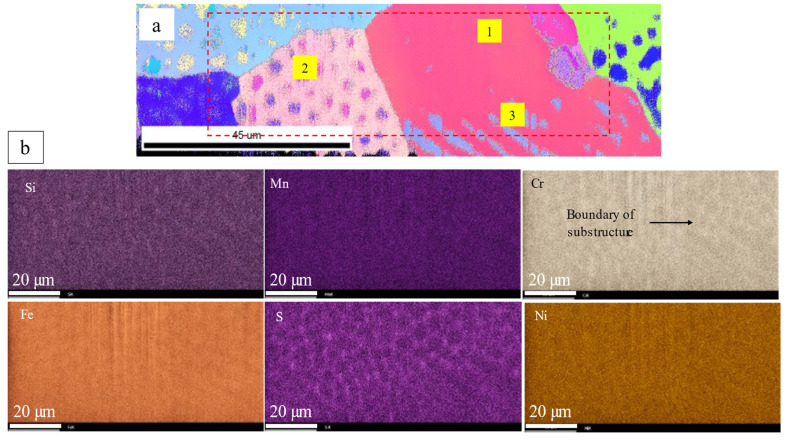
Chemical analysis of dual-phase structures in as-printed AISI 316L SS. (**a**) EBSD mapping. Grain boundary, substructure boundary and phase boundary are labeled. 1 and 3 indicate the single- and dual-phase structure of a grain correspondingly. Grain 2 indicates the cellular type dual phase structure. (**b**) EDS mapping of the region labeled by red box. Si, Mn, S, Cr and Ni were enriched, while Fe was depleted along the substructure boundary.

**Figure 5:**
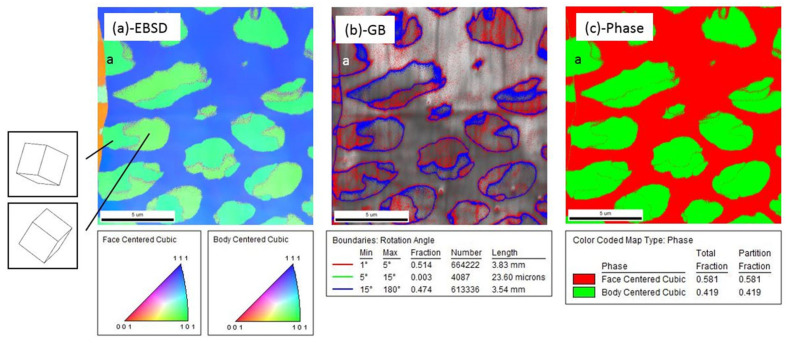
(**a**) Orientation imaging microscopy (OIM) of dual-phase structures. (**b**) Grain misorientation angle distribution. (**c**) Phase identification of dual-phase structures. Intercellular FCC-structured austenite and cellular BCC ferrite were observed. Grain with label “a” indicates a cellular structure that locates on the grain boundary with BCC structure but different grain orientations.

**Figure 6:**
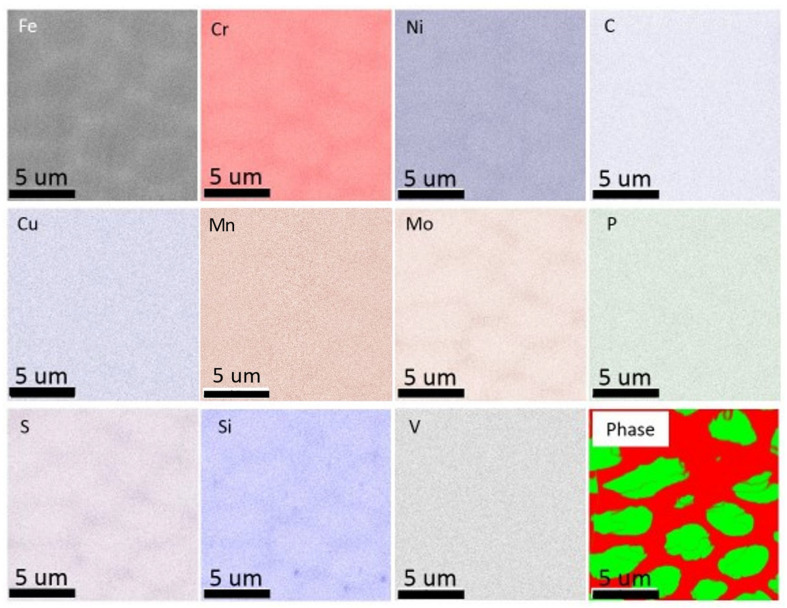
Chemical partitioning in dual-phase structures. EDS map reveals that Si, Mo, S, Cr and Ni were enriched and Fe was depleted in the austenite phase. Si, Mo, S, Cr and Ni were depleted, and Fe was enriched in the ferrite phase.

The chemical analysis of as-printed AISI 316L SS was performed using EDS. Figure 3 shows the chemical distribution in a region with single-phase structures, and the same region was examined for both OIM and chemical mapping. The dashed lines represent the grain boundaries. Chemical mapping shows that Si, Mo, S, Cr and Ni were enriched and Fe was depleted along the substructure boundary, and no clear evidence of chemical segregation was observed along the grain boundaries. Figure 4 shows the chemical distribution of a region with dual-phase structures. Elements enriched in fcc-structured austenite were Si, S, Cr, Co, Ni and Mo. Figure 5 shows the co-existence of fcc-structured austenite and bcc-structured ferrite in dual-phase structures. Bcc-structured ferritic grains in Figure 5a contained multiple subgrains with high-angle subgrain boundaries, as seen in Figure 5c. Chemical segregation was also related to phase separation in the as-printed AISI 316L SS. EDS chemical mapping of the same area in Figure 5 is shown in Figure 6. The result indicates that Fe was enriched in bcc-structured ferrite, and other elements, including Cr, Ni, C, Mn, Mo, S, Si, were enriched in fcc-structured austenite. 

### 3.2. In Situ Micro-Compression Tests

**Figure 7:**
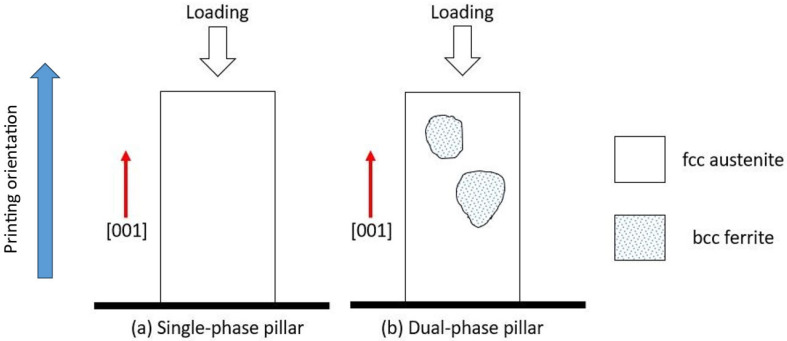
Sketch indicates the distribution of phases in micro-pillars. (**a**) Single-phase pillars. (**b**) Dual-phase pillars.

**Figure 8:**
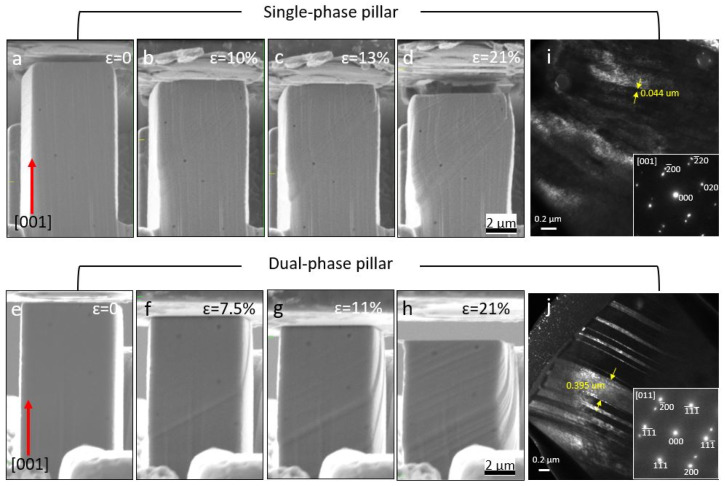
In situ SEM micro-compression of homogenous and dual-phase structures along the [001] orientation at room temperature. (**a**–**d**) Snapshots of compression tests of single-phase structures at strain rates of 0, 10%, 13% and 21%. (**e**–**h**) Snapshots of compression tests of dual-phase structures at strain rates of 1%, 7.5%, 11% and 21%. (**i**,**j**) TEM micrographs of compressed pillars showing the formation of deformation twins.

**Figure 9:**
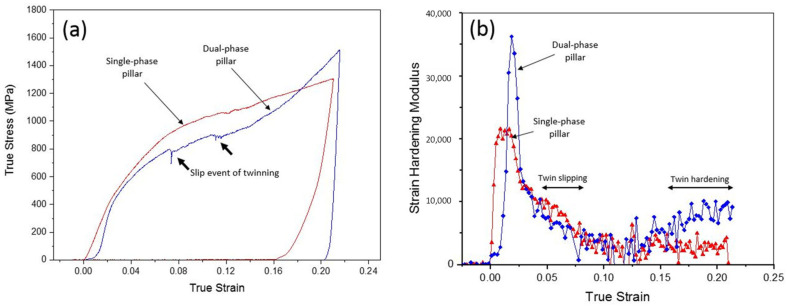
(**a**) True strain–stress curves of single-phase structures (red) vs. dual-phase structures (blue) of additively manufactured AISI 316L SS along the [001] orientation. The yield strength values were ~428 MPa for single-phase structures and ~418 MPa for dual-phase structures, and the Young’s moduli were ~21 GPa and ~38 GPa for single-phase structures and dual-phase structures, respectively. Two clear load drops were observed in the dual-phase structure. (**b**) Strain-hardening rates of single-phase structures and dual-phase structures as a function of true strain. Strain hardening induced by deformation twinning was observed in dual-phase structures when the true strain was greater than 0.12. Five pillars were tested in total, and the corresponding stress–strain plots are attached in the Supplemental Materials.

In situ micro-compression tests were performed in both single-phase and dual-phase structures along the [001] orientation at room temperature. Figure 7 sketches the distribution of phases and grain boundaries in both single-phase and dual-phase pillars. Figure 8 shows the snapshots of the micro-pillar compression tests at strain levels up to 21%. The snapshots suggest that twinning happened more obviously in dual-phase pillars than single-phase pillars. The TEM micrographs in Figure 8i,j confirm the formation of deformation twins in single-phase and dual-phase structures, but the twin spacing in deformed single-phase structures was ~44 nm, which was smaller than that in the deformed dual-phase structures, ~395 nm. The true stress–strain curves and corresponding strain-hardening rate as a function of strain are shown in Figure 9. The yield strength of single-phase structures was measured to be ~428 MPa, and that of dual-phase structures, ~418 MPa. The measured Young’s moduli were ~21 GPa for single-phase structures and ~38 GPa for dual-phase structures. Two clear load drops were observed during the compression of dual-phase structures, corresponding to the formation of deformation twins. The plot of the strain-hardening rate vs. strain indicates that twinning-induced strain hardening in dual-phase structures started when the true strain was greater than 12%. 

## 4. Discussion

### 4.1. Microstructure Evolution in As-Printed AM316L

**Figure 10:**
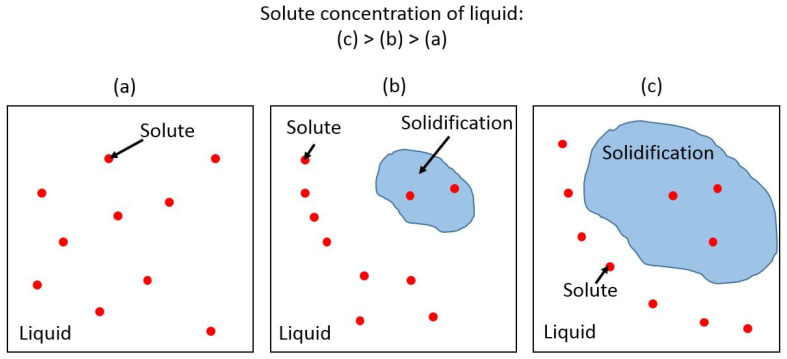
Sketch of elemental segregation during solidification. (**a**) Solute in 100% Liquid. (**b**) Solute in liquid and small amount of solidification. (**c**) Solute in liquid and large amount of solidification.

The thermal cycle in the local areas during EBM can generate metastable microstructures and non-uniform chemical distributions [39,40]. The size of the substructures (~5 µm) observed in LMD AM316L is much smaller than the grain size (~70 µm). Compared with the particle size of the powder used for printing (~40 µm), the size of the substructures is much smaller than either the grain size or powder particle size. Increased cooling rates and the corresponding non-equilibrium solidification in the SLM method are usually attributed to the cellular or columnar substructure according to previous research [41]. The size of the substructure is determined by energy input effects, including preheating temperature and scanning speed. Specifically, cell spacing decreases with the increase in laser scanning speed and the decrease in preheating temperature [42]. The formation of the cellular substructure requires a minimized cooling rate. The liquid solute concentration usually has to be minimized with an extremely high velocity of solidification at the front [43]. However, the solidification process was studied in Al-12Si systems [44], and it was shown that the solidifying front rejects solute back into the liquid under a high cooling rate, which increases the solute concertation at the solidifying front as solidification continues. The process is explained in the sketch in Figure 10. At the same time, solubility decreases as the temperature goes down, which makes elements segregate on the boundary of solidification. In the case of AM, the high-cooling-rate process is spread to the whole structure with much higher volume resolution than casting. Due to the combination of high cooling rate and changing solubility, the cellular structure is favored in kinetics [44]. The residual solute is segregated along the boundaries of the cellular substructure. A very similar process happened here in AM316L, and that explains the enrichment of solutes elements (Si, Mo, S, Cr, Ni, C, Mn and Co) and the depletion of Fe in the continuous phase. 

By combining the process of solidification, the evolution of the microstructure in LMD-processed AM316L, which is composed of both single-phase cellular–columnar austenite microstructure and dual-phase intercellular austenite microstructure, was explained by previous research on the relationship of electron-beam travel speed (cooling rate) and composition map in the welding process of Fe-Ni-Cr alloys by using an electron-beam as the heat source [43,45]. Research proved the strong effect of electron-beam travel speed, which is also the cooling rate, and the concentration of Ni and Cr on the microstructure. The result can give guidance on the relationship between the travel speed of the heat source/cooling rate and composition even though the heat source used in this research was different (laser beam). In the single-phase region, the majority of the microstructure is single-phase cellular–columnar austenite. In the dual-phase region, the formation of intercellular austenite with cellular ferrite comes from the combination effect of elemental segregation and change in the cooling rate. 

### 4.2. Effect of Dual-Phase Structures on Deformation Behavior in Additively Manufactured 316L

According to the contrast change of the micro-pillars during compression tests, deformation twinning is the major deformation mode for both single-phase and dual-phase structures, which can be observed from the diffraction patterns of both TEM lamellas of compressed pillars (Figure 8i,j). Even though the yield strength of single-phase structures was measured to be ~428 MPa, and that of dual-phase structures, ~418 MPa, the two structures are considered to have similar yield strength considering that the difference is so small. The two clear load drops are attributed to the formation of twins, as indicated in the TEM micrographs. The stress–strain plots of micro-compression testing (Figure 9a) reveal the different behavior of two pillars during compression in detail. The behavior was analyzed in elastic and plastic regimes, which include the 1st and 2nd load drops in the plastic regimes of the two pillars. In the elastic regime, the hardening effect of dual-phase-[001] pillars on yield strength was not obvious compared with that of single-phase-[001] pillars. When compression passed the yield point, twinning formed in hetero-[001] pillars, and the slip event from the formation of twins induced a load drop that could be observed with in situ compression and is indicated by arrows in the stress–strain plot in Figure 9a. 

In the review article by Meyers et al. [46], twinning is a two-fold effect on the evolution of plastic deformation: (1) Twinning contributes to plastic deformation with twinning shear, and twinning induces a decrease in the work-hardening rate. This has been found in Cu alloys by Vöhringer et al. [47]. (2) The grains are subdivided in twin grains, which drives the barriers to slip and increases the corresponding work-hardening rate. This has been demonstrated by Mulford and Kocks [48] and successfully modeled by Asgari et al. [49] and El-Danaf et al. [50]. Both effects of twinning can be observed in hetero-[001] pillars compared to homo-[001] pillars from their plots of strain hardening–strain in Figure 9b: In the twinning slipping period (strain = 0.037 to 0.07), the strain hardening of hetero-[001] pillars was lower than that of homo-[001] pillars due to twin-grain nucleation. When twin formation finished, the twin hardening effect started to show (strain > 0.14).

It is widely known that 1/6<112> Shockley partial dislocation creates the deformation twinning and stacking faults in fcc materials [51,52,53,54]. The preference for twinning and the effect of grain boundaries have been proved by previous research on nanocrystalline Al [55]. Research on nanocrystalline Al shows the strong effect of decreasing the grain size on initiating 1/6<112> Shockley partial dislocation and enhancing deformation twinning, where twin formation is usually difficult to observe in normal Al. Considering that both Al and AM316L are fcc-based materials, the effect of grain boundaries on enhancing deformation twinning should also be applicable in the AM316L used in this paper. The mechanism explains the formation of larger twins in dual-phase pillars, which is due to grain boundaries, than in single-phase pillars without grain boundaries. 

Beside the effect of grain size on deformation twinning, the chemical-segregation-induced change in SFE is another potential dominating factor of deformation twinning to explain the formation of obviously thick twins in dual-phase pillars. Previous research built a system to calculate SFE as a function of alloying. In the model by L. Vitos et al. [56], the effect of Cr and Ni on SFE was computed and compared with previous experimental results. The model shows that SFE always decreases with the increase in Cr, while SFE increases with the increase in Ni. However, the increasing tendency of Ni with SFE starts slowing down when Ni is beyond 12 at.%. 

For the AM316L SS used in pillar compression, elemental segregation was slightly different in single-phase and dual-phase regions: In single-phase regions, Cr was enriched from ~16 to ~18 at.%, and Ni was slightly enriched from ~13 to ~15 at.% at the intercellular position. Both tendencies indicate that the SFE of single-phase regions was ~30 mJ/m^2^. In dual-phase regions, Cr was enriched from ~17 to ~24 at.% and Ni was enriched from ~10 to ~12 at.% at the intercellular position. For single-phase regions, the enrichment of both Cr and Ni did not obviously change SFE. However, for dual-phase regions, the enrichment of Cr decreased SFE from ~33 to ~15 mJ/m^2^, and the enrichment of Ni increased SFE from ~22 to 30 mJ/m^2^, which indicates that the overall SFE was obviously decreased below 30 mJ/m^2^ by Cr enrichment. The difference in the SFE of single-phase and dual-phase regions explains the difference in deformation twinning in single-phase and dual-phase pillars: the deformation twins in single-phase pillars are uniform, similar and thin in thickness, as SFE is uniformly distributed in single-phase pillars. For dual-phase pillars, the intercellular region has lower SFE than the cellular region, which makes it easier for the region to form twins. Considering that the dual-phase pillar has a structure with lower SFE but more defects and phase boundaries, the mechanical behavior is intended to be harder in elastic deformation, as the dislocation could be blocked by defects, but also to be weaker in yield point, due to the microstructure having lower SFE. The extra phase boundaries in dual-phase pillars tend to concentrate stress during compression compared with single-phase pillars. 

## 5. Conclusions

AISI 316L SS was fabricated with the laser metal deposition technique. Both single-phase (fcc-structured austenite) and dual-phase (bcc-structured ferrite and fcc-structured austenite) structures were developed due to the non-uniform cooling rate during the manufacturing process. Substructures were formed within the grain interiors with enrichment of Si, Mo, S, Cr and Ni, and depletion of Fe along the substructure boundaries. A non-uniform cooling rate increases the possibility of forming dual-phase structures, which could degrade the mechanical stability of the component fabricated using laser metal deposition. The dual-phase structures exhibited increased Young’s modulus and decreased yield strength. Twinning was the dominant deformation mechanism of dual-phase structures at room temperature. Our study provides new insights into the relationship between the microstructure and mechanical properties of additively manufactured metallic materials, which suggests that removing elemental segregation and grain distortion are the potential future directions for utilizing laser metal deposition additive manufacturing in industrial applications.

## Figures and Tables

**Table 1:** Chemical analysis of AM316L specimen.

Element	Result wt%	Min wt%	Max wt%
Cr	16.23	16.00	18.00
Ni	13.07	10.00	14.00
C	0.025	0.000	0.030
Mn	0.31	0.000	2.00
P	<0.010	0.000	0.045
S	0.011	0.000	0.030
Si	0.73	0.000	1.00
Mo	2.09	2.00	3.00
Fe	Balance	Balance	Balance

## Data Availability

Data can be provided upon request from corresponding author.

## Supplementary Materials

The following supporting information can be downloaded at: https://www.mdpi.com/article/10.3390/ma16175980/s1, Figure S1: Calculation of porosity in four regions and the corresponding results; Figure S2: Stress–strain plots of all tested single-phase (homo-001) and dual-phase (hetero-001) pillars; Figure S3: EDS scanning of dual-phase region, which is same as the region in Figure 5. Blue and yellow regions overlapped the martensite phase (intercellular position), and the red region overlapped the ferrite region (cellular region). In the dual-phase region, Cr was enriched from ~17 to ~24 at.%, and Ni was enriched from ~10 to ~12 at.% at the intercellular position; Figure S4: EDS scanning of dingle-phase region. Line scanning result shows that Cr was enriched from ~16 to ~18 at.% and Ni was slightly enriched from ~13 to ~15 at.% at the intercellular position; Figure S5: EBSD mapping of the three orientations of printed cube. (a) Mapping of xz surface (normal to y direction). (b) Mapping of yz surface (normal to x direction). (c) Mapping of xy surface (normal to z direction). Figure S6: Original mapping of Figure 4 at higher resolution; Figure S7; X-ray micro-CT characterization of pores in as-printed AISI 316L SS. One large pore with a diameter of ~48 µm is shown.
