# Supplementary material for: Investigation of Deformation Behavior of Additively Manufactured AISI 316L Stainless Steel with In Situ Micro-Compression Testing
Source: Materials (Basel). 2023 Aug 31;16(17):5980. doi: 10.3390/ma16175980 (PMC10488655; doi:10.3390/ma16175980)
Supplement: Supplementary file 1 [file materials-16-05980-s001.zip › materials-2475084-supplementary.pdf]

Investigation of Deformation Behavior of Additive Manufactured AISI 316L Stainless Steel by In-Situ Micro-Compression Testing

Supplemental Materials

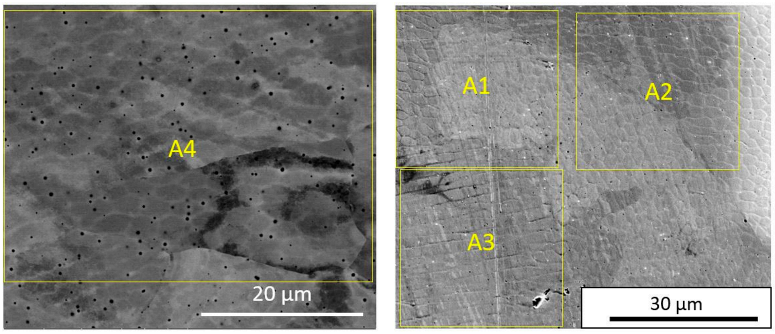

Area ID	Count	Average Single Pore Area (µm^2) / Average Size (µm)	Porosity (%)
A1	315	0.012 / 0.062	0.418
A2	81	0.053 / 0.129	0.989
A3	46	0.083 / 0.162	1.257
A4	71	0.069 / 0.148	1.170
Average	128.25	0.0543 / 0.132	0.9585

Figure S1. Calculation of porosity on four regions and the corresponding result.

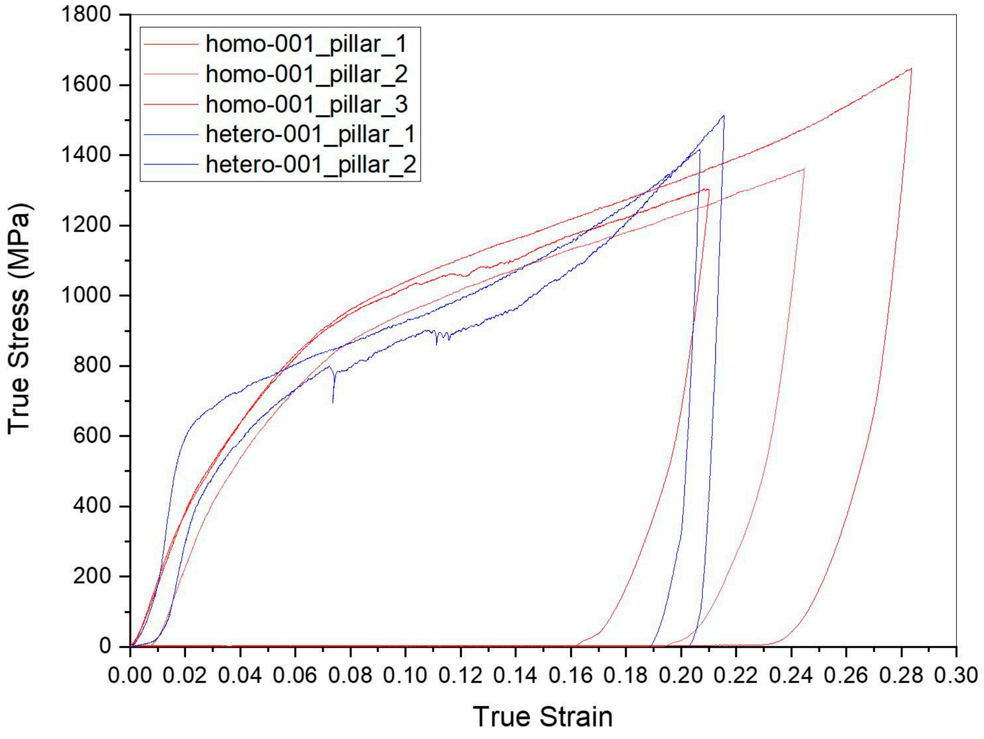

Figure S2. Stress-strain plots of all tested single-phase (homo-001) and dual-phase (hetero-001) pillars.

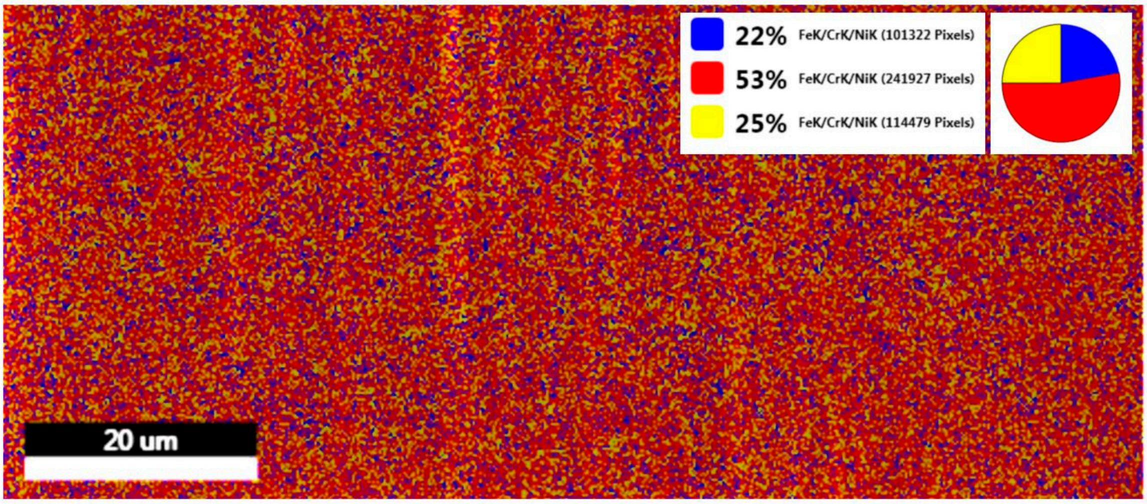

Red Spectrum

Element	Weight %	Atomic %
C K	0.80	3.61
SiK	0.00	0.00
P K	0.00	0.00
S K	0.18	0.30
CrK	16.96	17.58
MnK	0.91	0.89
FeK	69.04	66.65
CoK	1.74	1.59
NiK	9.94	9.13
MoK	0.43	0.24

Blue Spectrum

Element	Weight %	Atomic %
C K	0.95	4.24
SiK	0.01	0.03
P K	0.00	0.00
S K	0.27	0.44
CrK	23.38	24.01
MnK	0.73	0.71
FeK	62.07	59.35
CoK	1.99	1.80
NiK	9.99	9.08
MoK	0.61	0.34

Yellow Spectrum

Element	Weight %	Atomic %
C K	1.20	5.32
SiK	0.08	0.15
P K	0.00	0.00
S K	0.41	0.68
CrK	18.67	19.08
MnK	1.32	1.28
FeK	61.60	58.63
CoK	2.30	2.07
NiK	13.63	12.34
MoK	0.78	0.43

Figure S3. EDS scanning on dual-phase region, which is same as the region of Figure 5. Blue and yellow region overlaps the martensite phase (intercellular position) and red region overlaps the ferrite region (cellular region). In dual-phase region, Cr is enriched from ~17 to ~24 at.% and Ni is enriched from ~10 to ~12 at.% at intercellular position.

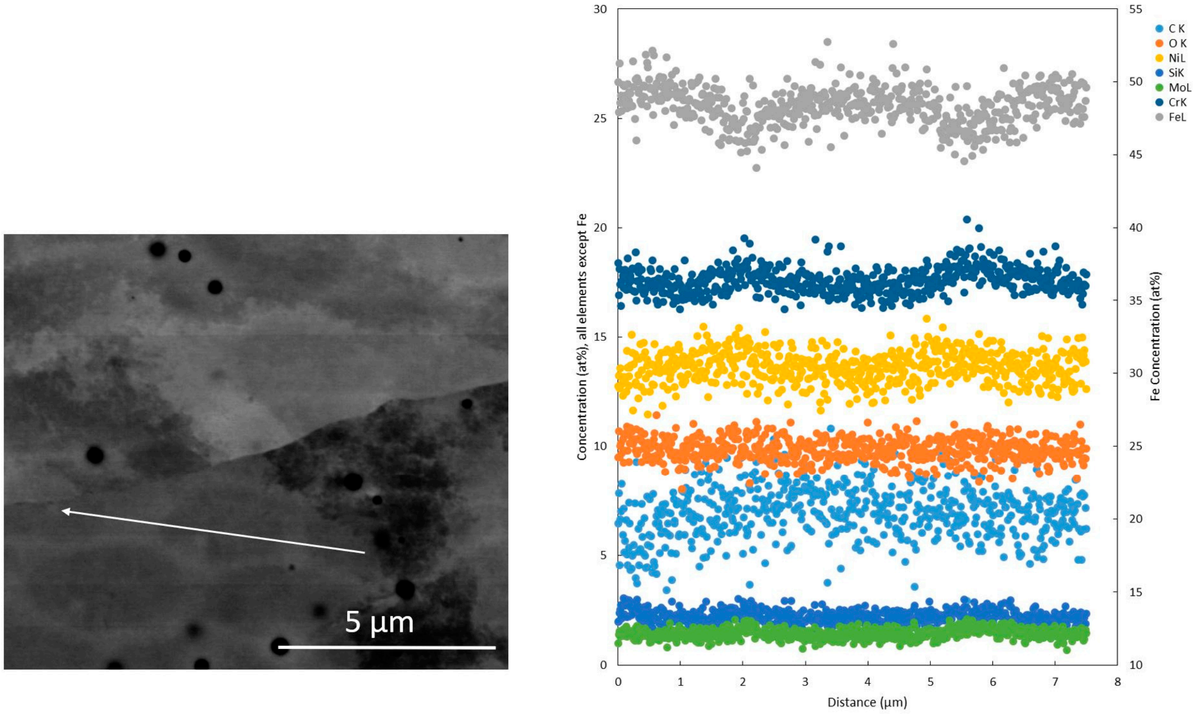

Figure S4. EDS scanning on dingle-phase region. Line scanning result shows that Cr is enriched from ~16 to ~18 at.% and Ni is slightly enriched from ~13 to ~15 at.% at intercellular position.

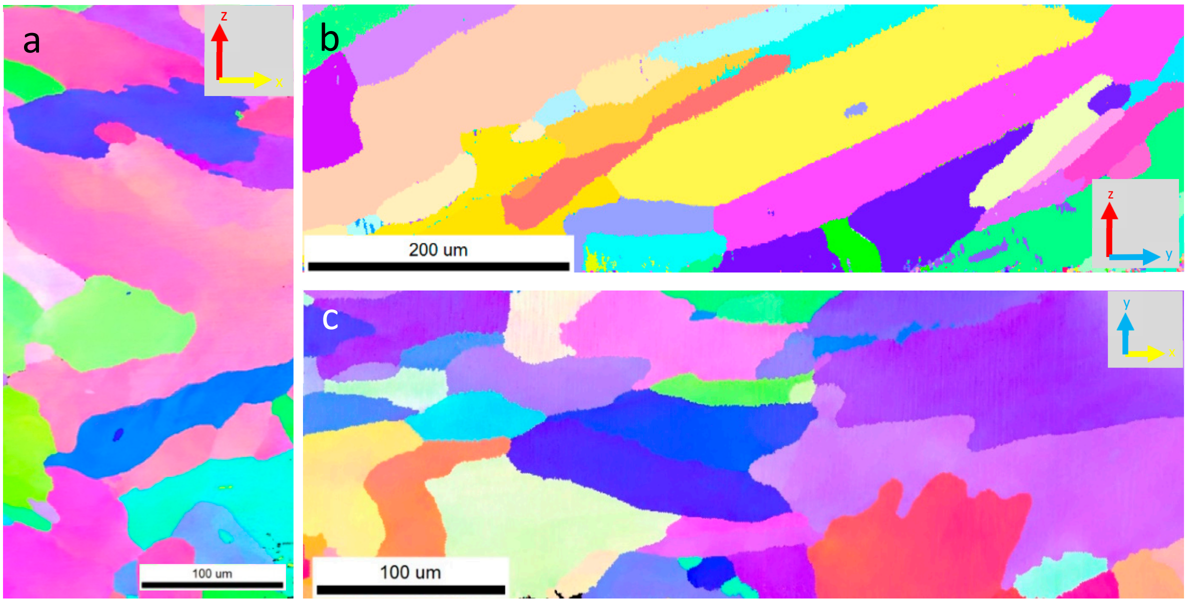

Figure S5. EBSD mapping of the three orientations of printed cube. (a) Mapping of xz surface (normal to y direction). (b) Mapping of yz surface (normal to x direction). (c) Mapping of xy surface (normal to z direction).

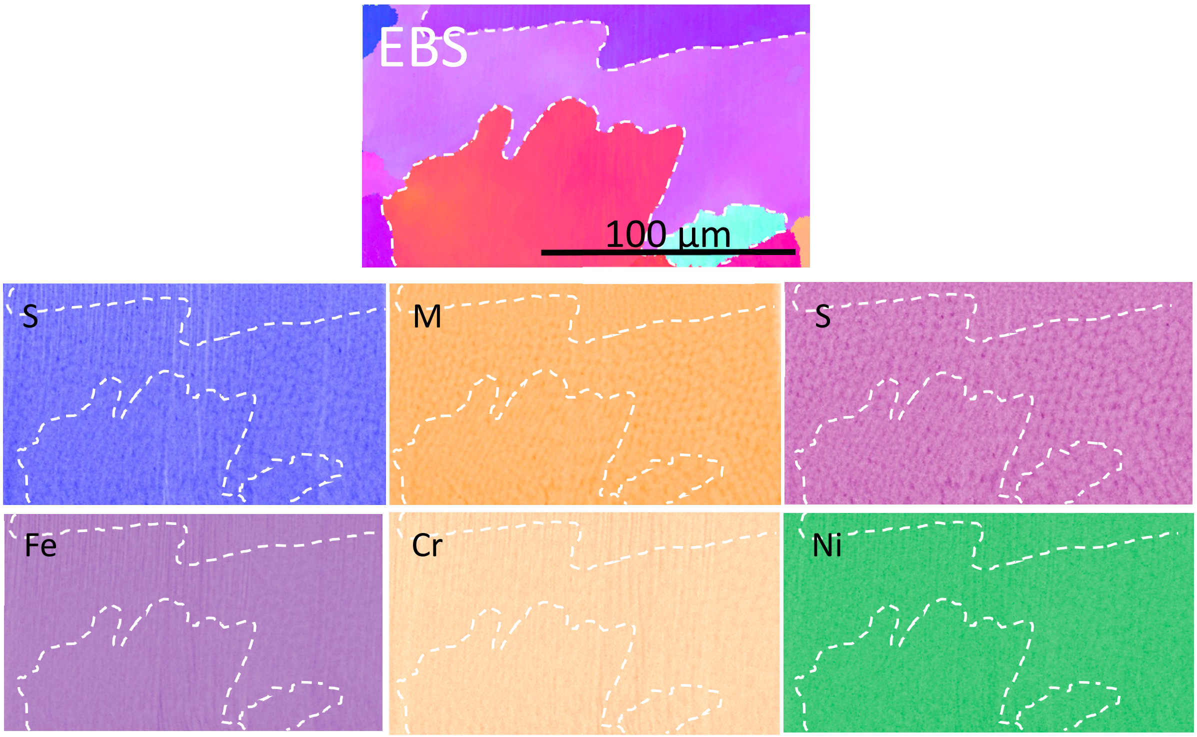

Figure S6. Original mapping of Figure 4 with higher resolution.

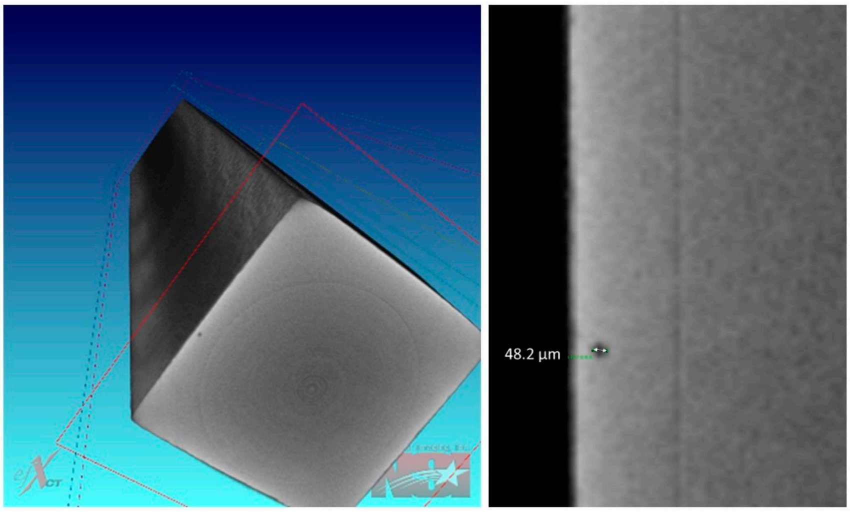

Figure S7. X-ray micro-CT characterization of pores in the as-printed AISI 316L SS. One large pore with diameter of ~ 48μm is shown.
